# Integrated postdischarge transitional care in a hospitalist system to improve discharge outcome: an experimental study

**DOI:** 10.1186/1741-7015-9-96

**Published:** 2011-08-17

**Authors:** Chin-Chung Shu, Nin-Chieh Hsu, Yu-Feng Lin, Jann-Yuan Wang, Jou-Wei Lin, Wen-Je Ko

**Affiliations:** 1 Department of Traumatology, National Taiwan University Hospital, No. 7, Chung-Shan South Road, Taipei 100, Taiwan; 2 Department of Internal Medicine, National Taiwan University Hospital, No. 7, Chung-Shan South Road, Taipei city 100, Taiwan; 3 Department of Internal Medicine, National Taiwan University Hospital, Yun-Lin Branch, No. 579, Yun-Lin Road, Douliou city, Yun-Lin county 640, Taiwan; 4 Department of Surgery, National Taiwan University Hospital, No. 7, Chung-Shan South Road, Taipei city 100, Taiwan

## Abstract

**Background:**

The postdischarge period is a vulnerable time for patients, with high rates of adverse events that may cause unnecessary readmissions, especially in the elderly. Because postdischarge care continuity is often interrupted after hospitalist care, close follow-up may decrease patient readmission. In this study, we aimed to investigate the impact of a quality improvement program, integrated postdischarge transitional care (PDTC), in Taiwan's hospitalist system.

**Methods:**

From December 2009 to May 2010, patients admitted to the hospitalist ward of a medical center in Taiwan and later discharged alive to home care were included. Efforts to improve the quality of interventions in the PDTC program, including a disease-specific care plan, telephone monitoring, hotline counseling and referral to a hospitalist-run clinic, were implemented in the latter four months in the intervention group, while the control group was recruited during the first two months of the study period. The primary end point was unplanned readmission or death within 30 days after discharge.

**Results:**

There were 94 and 219 patients in the control and intervention groups, respectively. Both groups had similar characteristics at the time of admission and at discharge. In the intervention group, 18 patients with worsening disease-specific indicators recorded during telephone monitoring and 21 patients with new or worsening symptoms recorded during hotline counseling had higher rates of unplanned readmission than those without worsening disease-specific indicators (*P *= 0.031) and worsening symptoms (*P *= 0.019), respectively. Patients who received PDTC had lower rates of readmission and death than the control group within 30 days after discharge (15% vs. 25%; *P *= 0.021). Nonuse of a hospitalist-run clinic and presence of underlying malignancy were other independent risk factors for readmission and death within 30 days after discharge.

**Conclusion:**

Integrated PDTC using disease-specific care, telephone monitoring, hotline counseling and a hospitalist-run clinic can reduce rates of postdischarge readmission and death.

## Background

The hospitalist system has grown worldwide in recent decades [1-3], even though its pros and cons are still a matter of debate. While the hospitalist system may lessen hospitalization costs, interruption of patient care provided by the primary care physician is a major concern [4]. In fact, short-term postdischarge readmission rates are very high in the elderly, approaching 20% within one month after discharge in a US analysis [5]. The reasons for high readmission rates include poor compliance, instability of chronic disease and insufficient communication between inpatient and outpatient physicians [6]. Home visits and telemedicine have been studied in postdischarge care, but the reported effectiveness is limited to those with congestive heart failure or chronic obstructive pulmonary disease as well as patients who have undergone surgery [7-11].

In our referral center in Taiwan, the one-month readmission rate after discharge from the hospitalist ward is 22% (unpublished data, C.C.S. and W.J.K.). Based on the concept of Care Transitions Intervention [12], postdischarge transitional care (PDTC) is important in extending the continuity of care after discharge [13,14]. Close follow-up and communication may prevent adverse events and decrease readmission rates before the primary care physician takes over care continuity [15].

Although experience with PDTC has been studied extensively, the effectiveness of an integrated model using telephone service in a hospitalist system has not been well documented [8,12,16]. Given the success of Care Transitions Intervention in decreasing postdischarge adverse events and reducing readmission rates [12], in this study we aimed to investigate whether a quality improvement program for PDTC consisting of a disease-specific care plan established at discharge, a patient hotline, scheduled follow-up calls and a hospitalist-run clinic could reduce readmission rates and postdischarge mortality in a hospitalist care system.

## Methods

### Study subjects

This prospective experimental study was conducted at the National Taiwan University Hospital, a tertiary care referral center in northern Taiwan. The hospital's Institutional Review Board approved the study (200900012023R). From December 2009 to May 2010, we consecutively screened all patients older than 18 years of age who had been admitted to the hospitalist care ward from the emergency department (ED). Those who were discharged alive to home were enrolled and grouped according to the designated general medical diseases (Table 1). The disease-based subgroups were monitored by using specific care plans. Other inclusion criteria included a telephone line at home and a caregiver or patient who could speak Chinese or Taiwanese.

**Table 1:** Disease-specific indicators designated for postdischarge care by telephone call follow-up^a^

Disease	Indicator 1	Indicator 2	Indicator 3
Chronic disease with acute change			
CHF with acute exacerbation	Body weight	Leg edema^b^	Dyspnea index^c^
Liver cirrhosis with decompensation	Body weight	Consciousness	
COPD with acute exacerbation	Fever	Dyspnea index^c^	Sputum character
DM with poor control	Blood glucose		
Hypertension with poor control	Blood pressure		
Acute on chronic renal failure	Body weight	Urine output	
Terminal cancer	Consciousness	Pain scale^d^	Dyspnea index^c^
Acute illness			
Ischemic stroke	Barthel score	Consciousness	
UGI bleeding	Stool character	Heart rate	
Pneumonia	Fever	Dyspnea index^c^	
Urinary tract infection	Fever	Dysuria	
Cellulitis	Size of lesion	Local pain^d^	Fever
Intraabdominal infection	Fever	Abdominal pain^d^	

^a^CHF, congestive heart failure; COPD, chronic obstructive pulmonary disease; DM, diabetes mellitus; UGI, upper gastrointestinal. Blood pressure, body temperature, heart rate and blood glucose were measured by the patients or their caregivers at home. ^b^Measured by grading developed for cancer treatment [26]. ^c^Measured according to the Medical Research Council dyspnea scale [27]. ^d^Measured according to the Numerical Rating Scale [28].

Patients were excluded if they were electively admitted, died during hospitalization, were transferred to a subspecialty ward or other institutions or refused to provide consent. Patients without an underlying chronic illness and with a Barthel Index score > 60 were also excluded because they presumably did not require monitoring [17].

For a quality improvement initiative, during the first two months of the study period, the patients received no active intervention except for a follow-up call 30 days after discharge to confirm the patient's status regarding readmission, ED visit and survival (control group). In the latter four months of the study period, the patients received integrated PDTC for 30 days after discharge (intervention group).

For every patient, the hospital's doctors created a postdischarge care plan. The ward staff educated the patients and their caregivers regarding this postdischarge care plan, which usually included chest care, inhaler use, tube management and wound care skills, diet and drug compliance, and other disease-specific elements. Patients were interviewed before discharge to screen them for their language ability and cognition. A patient was considered to have a cognition deficit if orientation, attention and recall ability were not intact [18]. Caregivers were given the postdischarge instructions if the patient had a language proficiency limitation, cognitive deficits or a Barthel Index score < 35.

The patients were referred back to the clinic of their primary care physician for continuity of care. A physician was considered to be the patient's primary care physician if the patient had visited the same doctor three times or more within one year prior to admission [4]. Patients who had no primary care physician were followed up at the hospitalist-run clinic.

### Integrated PDTC protocol

For the intervention group in the quality improvement program, the study nurses and hospital physicians gave patients a PDTC plan that consisted of a disease-specific care plan, scheduled follow-up calls and a hotline to monitor their disease status and their disease-specific indicators (Table 1). The PDTC plan was added onto the usual discharge care plan. The disease-specific indicators were initially chosen and then modified by the hospital physician according to the patient's condition [19]. The study nurses and physicians educated the patients and their caregivers regarding measuring and reporting the disease-specific indicators and adhering to the postdischarge care plan and medication use.

After discharge, the study nurse contacted the patients regularly by telephone on postdischarge days 1, 3, 7, 14 and 30. Using a standardized case report form, the content of telephone calls included (1) monitoring disease-specific indicators, (2) enhancing drug compliance and (3) confirming adherence to the postdischarge care plan, including diet and lifestyle modification as well as tube management and wound care skills. A designated telephone line was also opened from 8:00 AM to 9:00 PM daily for call-in counseling for the intervention group.

Once disease worsening was noted (Additional file 1), the study nurses reported this to the patient's hospitalist and discussed further management, including counseling and referral to the clinic or the ED. The hospitalist-run clinic was open from 8:00 AM to 9:00 PM daily and was managed by a hospitalist.

### Clinical characteristics

The study patients' clinical characteristics, laboratory data and hospital course were collected by board-certified nurses who were blinded to the study. We used a unified case report form which contained a default option for selection to avoid ambiguous data coding. The κ agreement was excellent in testing. The Charlson Index and Barthel Index scores were calculated as outlined in previous studies [20,21]. Patients were followed up for 30 days after discharge or until they died, were readmitted or were lost to follow-up. A patient was considered lost to follow-up if the patient or caregiver could not be contacted two consecutive times. The study team had weekly meetings to confirm patients' status codes and monitor PDTC implementation, including screening, enrollment, follow-up and termination of patient care.

The primary end point was unplanned readmission or unexpected death within 30 days after discharge. Secondary end points included visits to the ED or the hospitalist-run clinic. An urgent or unplanned clinic visit was defined as one that occurred < 24 hours from an appointment to the clinic visit.

### Statistical analysis

Intergroup differences were compared by performing an independent *t*-test for numerical variables and a χ^2 ^test for categorical variables. Curves representing the probability of readmission and death were generated using the Kaplan-Meier method and compared by using the log rank test. Factors associated with 30-day primary outcomes were identified by a forward conditional method using multivariate Cox proportional hazards regression. A two-sided *P *< 0.05 was considered significant. All analyses were performed using SPSS version 13.0 software (SPSS, Inc., Chicago, IL, USA).

## Results

**Figure 1:**
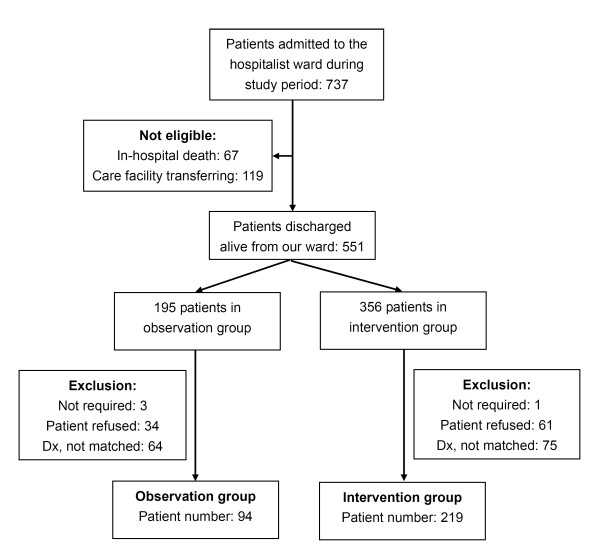
**Flowchart of patient enrollment**. "Not required" indicates a patient with no chronic illness and a Barthel Index score ≥60; "Dx, not matched" means the patient's diagnosis did not match the enrolled disease items; "Patient refused" means the patient refused enrollment.

From December 2009 to May 2010, 2,932 patients were admitted to the general ward from the ED. Of the 737 patients admitted to the hospitalist ward, only 551 were discharged alive for home care (Figure 1), among whom 139 patients did not match the designated general medical diseases for enrollment, 95 declined enrollment and 4 were defined as not requiring PDTC. Among the 313 patients finally enrolled, 94 were recruited into the control group and 219 into the intervention group.

The clinical characteristics of the patients were similar between the two groups, including age, gender, underlying comorbidities and the presence of a primary care physician (Table 2). The results of laboratory examinations conducted upon admission were also similar. Upon discharge, patients in the control group were more frequently cared for by their children (40% vs. 27%; *P *= 0.009), whereas those in the intervention group were more commonly cared for by their spouses (42% vs. 31%; *P *= 0.084).

**Table 2:** Clinical characteristics and laboratory data at initial admission compared between control and intervention groups

Clinical characteristics	Control group (*n *= 94)	Intervention group (*n *= 219)	*P *value
Mean age (± SD), years	71 ± 15	69 ± 16	0.207
Males, *n *(%)	42 (45%)	115 (53%)	0.204
Mean Charlson Index score (± SD)	3.5 ± 3.2	3.1 ± 3.1	0.210
Presence of primary care physician, *n *(%)	66 (70%)	173 (79%)	0.094
Underlying malignancy, *n *(%)	30 (32%)	57 (26%)	0.287
Mean laboratory data at initial admission^a ^(± SD)			
Leukocyte count, cells/μL	10,079 ± 5,011	10,903 ± 5,588	0.232
Hemoglobin, g/dL	13.2 ± 18.3	11.2 ± 2.6	0.228
Creatinine, mg/dL	2.6 ± 5.1	1.9 ± 2.0	0.284
Caregiver at home, *n *(%)			
Child generation	38 (40%)	58 (27%)	0.009
Parental generation	3 (3%)	5 (2%)	0.617
Spouse	29 (31%)	92 (42%)	0.084
Nonrelative caregiver	21 (22%)	62 (28%)	0.321
Mean Barthel Index score at discharge (± SD)	62 ± 35	66 ± 37	0.378
Mean length of hospital stay, days (± SD)	8.5 ± 8.0	8.9 ± 6.1	0.660
Artificial tube and/or catheter at discharge, *n *(%)	22 (23%)	51 (23%)	0.982
Wound needing dressing changes, *n *(%)	10 (11%)	27 (12%)	0.671

^a^Results of hemograms and renal function tests were not available for 23 and 25 patients, respectively.

Both groups had comparable activities of daily living assessments on the basis of Barthel Index scores, length of hospitalization and defined diseases (Additional file 1). The number of patients who needed artificial intubation (for example, nasogastric tube, tracheostomy tube and draining tube) and catheters (for example, Foley catheter, catheter for dialysis and draining tube), and thus required additional care, was similar between the two groups. Wounds, mostly bedsores that required regular cleaning and dressing at home, were noted in 11.8% of the study patients in both groups combined.

In the postdischarge course, 843 calls were recorded, with an average ± standard deviation of 6.10 ± 2.96 minutes per call. Among the 219 patients who received PDTC, 134 received all of the calls and PDTC for the remaining 85 was terminated earlier in the postdischarge period, comprising 53 who were lost to follow-up and 32 patients who were readmitted or died. Eighteen had worsening disease-specific indicators, comprising six who were referred to the ED and twelve who were referred for clinic appointments (only two attended the hospitalist-run clinic). Six patients (five in the ED and one in the clinic) were readmitted within 30 days after discharge. Those with worsening indicators had a significantly higher risk of readmission than those without them (6 (33%) vs. 26 (13%); *P *= 0.031, Fisher's exact test). During telephone contacts, four patients were found to be using the wrong tube or wound care techniques, and they were given immediate instructions pertaining to their home care. The subsequent telephone contacts confirmed that all of them had corrected the care techniques. During the telephone contacts, only two patients reported poor drug compliance, which improved after they received advice. These two incidents were not significantly associated with readmission.

Forty-four patients (20%) in the intervention group contacted our team a total of one hundred five times using the designated telephone line. Of these, 29 calls from 21 patients were made to report new or worsening symptoms. Four patients were referred to the ED, and eleven were referred to the outpatient clinic. The remaining six received counseling only. Seven patients (33%) were readmitted (four from the ED and three from the clinic). Those reporting new or worsening symptoms had a higher risk of readmission than those without these symptoms (7 (33%) vs. 25 (13%); *P *= 0.019, Fisher's exact test). All of the other 76 patients who called counseling asked for minor medical help, such as health education, skill confirmation and a drug or diet consultation.

In terms of postdischarge clinic appointments, there were more scheduled appointments at the hospitalist-run clinic, comprising either regular visits (25 (27%) vs. 31 (14%); *P *= 0.008) or unplanned visits (8 (9%) vs. 4 (2%); *P *= 0.005), and fewer with the primary care physician (68 (72%) vs. 181 (83%); *P *= 0.038) in the control group than in the intervention group. Visits to the hospitalist-run clinic were associated with fewer readmissions (*P *= 0.088) than no visits, whereas visits to the primary care physician clinic were not significant (*P *= 0.890). The number of ED visits was not different between the control and intervention groups (22 (23%) vs. 38 (17%); *P *= 0.212).

**Figure 2:**
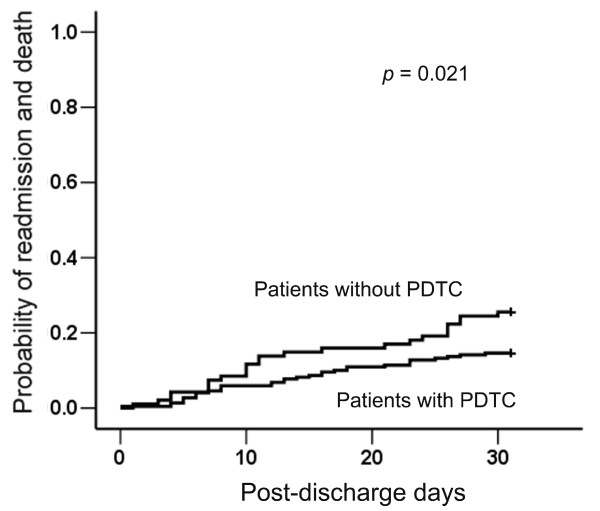
**The probability of readmission and unexpected death within 30 days after discharge was plotted using the Kaplan-Meier method and compared using the log rank test**. PDTC, postdischarge transitional care.

Within 30 days after discharge, the control group had significantly higher rates of readmission and death than the intervention group (24 (25%) vs. 32 (15%); *P *= 0.021, log rank test) (Figure 2). Further analysis revealed that the control group trended toward a higher readmission rate (21 (22%) vs. 31 (14%); *P *= 0.075) and a significantly higher death rate (3 (3%) vs. 1 (1%); *P *= 0.048). In contrast, the readmission rates of all patients in the general wards of the hospital were similar during the control and intervention periods (17.0% vs. 17.2%; *P *= 0.913, χ^2 ^test) (see Additional file 1).

Multivariate Cox proportional hazards regression revealed three independent factors related to primary outcome within 30 days after discharge, including underlying malignancy (hazard ratio (HR) 2.34, 95% confidence interval (95% CI) 1.33 to 4.11), not visiting the hospitalist-run clinic after discharge (HR 2.65, 95% CI 1.04 to 6.73) and not receiving PDTC (HR 2.05, 95% CI 1.16 to 3.65) (Table 3). Artificial tubes and catheters, wounds requiring dressing changes, age > 65 years, Barthel Index score < 60, visits to the primary care physician clinic and longer hospital stay (≥14 days) were not associated with postdischarge readmission or death.

**Table 3:** Multivariate analysis of factors possibly associated with readmission and unexpected death within 30 days after discharge^**a**^

Factors	Data	Multivariate analysis	
		
		*P *value	HR (95% CI)
Age, years	≥65	0.980	
	< 65		
Gender	Male	0.423	
	Female		
Artificial tube or catheter	At least one	0.880	
	None		
Wound needing dressing changes	Presence	0.404	
	Absence		
Charlson Index score	< 2		
	2 to 4	0.580	
	> 4	0.418	
Barthel Index score at discharge	< 60	0.208	
	≥60		
Primary care physician	Presence	0.710	
	Absence		
Underlying malignancy	Yes	0.003	2.34 (1.33 to 4.11)
	No		
Length of hospital stay	< 14 days	0.188	
	≥14 days		
Blood leukocyte count, cells/μL	6,000 to 11,000	0.494	
	< 6,000 or > 11,000		
Postdischarge transitional care	Not received	0.014	2.05 (1.16 to 3.65)
	Received		
Postdischarge disease type	Chronic illness	0.172	
	Acute illness		
Visits to hospitalist-run clinic	Not received	0.041	2.65 (1.04 to 6.73)
	Received		
Caregiver at home	Not spouse	0.465	
	Spouse		

^a^HR, hazard ratio; CI, confidence interval.

## Discussion

In this study, we investigated experiences with an integrated PDTC program consisting of a disease-specific care plan, follow-up phone calls, hotline counseling and referral to a hospitalist-run clinic to decrease postdischarge adverse events. This PDTC program implemented in a hospitalist ward in a Taiwan referral center significantly reduced readmission and mortality rates within 30 days after discharge. Aside from receiving integrated PDTC, the absence of underlying malignancy as well as visits to the hospitalist-run clinic were also associated with better outcomes.

The best method for transitional care after discharge is not well established, although several models have been studied, including telephone call follow-up, telehealth communication and monitoring and home visits by nursing staff [10,22-24]. An integrated PDTC program using telephone call follow-up is not difficult to implement and has minimal equipment requisites, though infrastructure and personnel are needed for proper and sustainable follow-up [23]. The telephone service in the current study includes active regular contact to detect disease worsening as soon as possible after discharge, as well as a standby counseling service. In addition, the service monitors patient medication compliance and lifestyle modifications. The standby counseling service is helpful, especially for care-related questions and unexpected events. However, the number of ED visits has not been significantly reduced, probably because the service is only available from 8:00 AM to 9:00 PM and some aspects of the ED are irreplaceable.

The findings of the current study suggest that outpatient clinics run by hospitalists may play an important role in improving postdischarge outcomes. Outpatient follow-up by a hospitalist who is familiar with patients' disease status can provide continuous care for discharged patients, and it complements PDTC. With the use of integrated PDTC, patients and physicians may have more confidence in the care provided, and the need for scheduled hospitalist-run clinic visits may thus be decreased [23]. PDTC can effectively reduce unplanned visits to the hospitalist-run clinic. For instance, because the control group in our study lacked a hotline or telephone contact service, the frequency of hospitalist-run clinic visits increased in those patients not receiving PDTC. In contrast, the frequency of such visits decreased in the intervention group.

However, the higher percentage of primary care physician postdischarge clinic follow-up in the intervention group compared to the control group might be another cause of the reduced visits to the hospitalist-run clinic. Nonetheless, hospitalist care after discharge is very important because approximately 20% to 30% of patients do not have primary care physicians. Transitional care indeed plays an important role in reducing readmission after discharge [12-14]. Increased communication with outpatient physicians who routinely cared for the patient before admission reportedly decreases the risk of urgent readmission [13,25]. Therefore, the continuity of postdischarge patient care can be achieved by integrated PDTC and hospitalist-run clinics.

### Limitations

This study has several limitations. First, we used a quality improvement initiative design without randomization. This may not be a serious concern because of the similarities of the clinical characteristics between the two study groups and the readmission rate of patients discharged from the general wards. Second, telephone monitoring may be biased by patients' or caregivers' incorrect statements. Third, the number of patients excluded was considerable and might also have biased the results. However, we made an effort to enroll a homogeneous cohort of patients from a general medical population admitted from the ED. More care should be exercised regarding excluding patient populations in the future. Last, because this study was performed in a tertiary care referral center and the patients had multiple comorbidities, the results cannot easily be generalized to regional or district hospitals.

## Conclusion

The hospitalist system has become widely accepted in recent decades, with advances developed to cope with any discontinuity between inpatient and outpatient care. However, hospitalist PDTC guidelines are still lacking. Apart from the discharge summary and postdischarge care planning, the current study shows that integrated PDTC is effective in providing transitional care and may be applied to general medical patient populations who require an ever-increasing amount of resources in an aging society.

## Competing interests

The authors declare that they have no competing interests.

## Authors' contributions

WJK designed the study. CCS, NCH and YFL were involved in project execution, clinical data collection, data analysis and the writing of the manuscript. JWL participated in data analysis. JYW contributed to the writing of the manuscript. All authors read and approved the final manuscript.

## Pre-publication history

The pre-publication history for this paper can be accessed here:

http://www.biomedcentral.com/1741-7015/9/96/prepub

## Supplementary Material

Additional file 1 **Figure S1**. The overall probability of readmission within 30 days from discharge for general medical patients admitted from the emergency department was plotted using the Kaplan-Meier method and compared using the log rank test. Table S1. Criteria for worsening of each disease-specific indicator. Table S2. Comparison of underlying diseases between the observation and intervention groups. Click here for file
