# Supplementary material for: Integrated postdischarge transitional care in a hospitalist system to improve discharge outcome: an experimental study
Source: BMC Med. 2011 Aug 17;9:96. doi: 10.1186/1741-7015-9-96 (PMC3170615; doi:10.1186/1741-7015-9-96)
Supplement: Additional file 1 — Figure S1. The overall probability of readmission within 30 days from discharge for general medical patients admitted from the emergency department was plotted using the Kaplan-Meier method and compared using the log rank test. Table S1. Criteria for worsening of each disease-specific indicator. Table S2. Comparison of underlying diseases between the observation and intervention groups. [file 1741-7015-9-96-S1.DOC]

**ONLINE SUPPLEMENT**

**Overall readmission rate in the general ward of the study hospital**

During the study period, 2932 patients were admitted to the general wards from the emergency department but 2393 were later discharged for home or to a nursing facility care. By classification, 854 patients were identified from December 2009 to January 2010 while 1539 were identified from February to May 2010. Their readmission number was 145 (17.0%) and 264 (17.2%), respectively. When plotted by the Kaplan Meier method (Fig. S1), the readmission rate was not different in the general wards even with seasonal changes.

**LEGEND**

**Figure S1.** The overall probability of readmission within 30 days from discharge for general medical patients admitted from the emergency department was plotted by the Kaplan Meier method and compared using the log rank test.

**Table S1. Criteria of worsening for each disease-specific indicator**

| Indicator | Worsening criteria |
| --- | --- |
| Barthel score | <60% of baseline [1] |
| Blood glucose | >400 mg/dL anytime or >250 mg/dL AC |
| Blood pressure | Systolic pressure >180 mm Hg, diastolic >105 mm Hg |
| Body weight | Weight gain ≥1Kg |
| Body temperature | ≥38℃ |
| Consciousness | decrease in Glasgow coma scale by >1 grade |
| Dyspnea**^†^** | Increase in dyspnea score by >1 grade |
| Heart rate | >120 bpm or <60 bpm |
| Leg edema* | Increase in edema score by >1 grade |
| Pain^#^ | Increase in pain scale by >2 or pain scale >4 |
| Stool | Black or bloody |
| Sputum | Bloody or purulent |
| Size of local lesion** | Increase in size by 20% |
| Urine output | <60% of baseline daily amount or 0.5ml/Kg/hr [2] |

*measured by a grading system developed for cancer treatment [3].

**^†^**measured by the Medical Research Council dyspnea scale [4].

^#^measured by the Numerical Rating Scale [5].

**measured by the longest length of the lesion.

**Table S2.** Comparison of underlying diseases between the observation and intervention groups

|  | Observation group (n=94) | Intervention group (n=219) | *p* value |
| --- | --- | --- | --- |
| Major disease entities for follow-up |  |  |  |
| Chronic illness with acute change | 32 (34) | 61 (28) | 0.142 |
| CHF with acute exacerbation | 5 (5) | 13 (6) | 0.830 |
| Liver cirrhosis with decompensation | 6 (5) | 11 (6) | 0.626 |
| COPD with acute exacerbation | 5 (5) | 14 (6) | 0.715 |
| DM with poor control | 9 (10) | 8 (4) | 0.034 |
| Hypertension with poor control | 2 (2) | 0 | 0.030 |
| Acute on chronic renal failure | 4 (4) | 12 (6) | 0.953 |
| Terminal cancer | 1 (1) | 3 (1) | 0.825 |
| Acute illness | 62 (66) | 158 (72) | 0.142 |
| Ischemic stroke | 1 (1) | 8 (4) | 0.209 |
| UGI bleeding | 7 (7) | 28 (13) | 0.169 |
| Pneumonia | 21 (22) | 34 (16) | 0.119 |
| Urinary tract infection | 23 (25) | 34 (16) | 0.060 |
| Cellulitis | 2 (2) | 14 (6) | 0.116 |
| Intra-abdominal infection | 8 (9) | 40 (18) | 0.444 |

Abbreviations: CHF, congestive heart failure; COPD, chronic obstructive pulmonary disease; DM, diabetes mellitus; UGI, upper gastrointestinal

Data are no. (%) or mean ± standard deviation unless otherwise indicated

**REFERENCES**

1. Leong IY, Chan SP, Tan BY, Sitoh YY, Ang YH, Merchant R, et al. Factors affecting unplanned readmissions from community hospitals to acute hospitals: a prospective observational study. *Ann Acad Med Singapore*. 2009; 38:113-20.

2. Jerng JS, Ko WJ, Lu FL, Chen YS, Huang SF, Yu CJ, et al. Incidence and significance of clinically abnormal events in a tertiary referral medical center: implementation of the clinical alert system (CAS). *J Formos Med Assoc*. 2008; 107:396-403.

3. Trotti A, Colevas AD, Setser A, Rusch V, Jaques D, Budach V, et al. CTCAE v3.0: development of a comprehensive grading system for the adverse effects of cancer treatment. *Semin Radiat Oncol*. 2003; 13:176-81.

4. Bestall JC, Paul EA, Garrod R, Garnham R, Jones PW, Wedzicha JA. Usefulness of the Medical Research Council (MRC) dyspnoea scale as a measure of disability in patients with chronic obstructive pulmonary disease. *Thorax*. 1999; 54:581-6.

5. Stinson JN, Kavanagh T, Yamada J, Gill N, Stevens B. Systematic review of the psychometric properties, interpretability and feasibility of self-report pain intensity measures for use in clinical trials in children and adolescents. *Pain*. 2006; 125:143-57.
